# Self-Rated Health in middle-aged and elderly Chinese: distribution, determinants and associations with cardio-metabolic risk factors

**DOI:** 10.1186/1471-2458-9-368

**Published:** 2009-09-29

**Authors:** Nazanin Haseli-Mashhadi, An Pan, Xingwang Ye, Jing Wang, Qibin Qi, Yong Liu, Huaixing Li, Zhijie Yu, Xu Lin, Oscar H Franco

**Affiliations:** 1 Unilever R&D, Colworth Science Park, Sharnbrook, Bedfordshire, MK441LQ, UK; 2 Key Laboratory of Nutrition and Metabolism, Institute for Nutritional Sciences, Shanghai Institutes for Biological Sciences, Chinese Academy of Sciences and Graduate School of the Chinese Academy of Sciences, Shanghai, PR China; 3 Health Sciences Research Institute, Warwick Medical School, The University of Warwick. Coventry CV47AL, UK

## Abstract

**Background:**

Self-rated health (SRH) has been demonstrated to be an accurate reflection of a person's health and a valid predictor of incident mortality and chronic morbidity. We aimed to evaluate the distribution and factors associated with SRH and its association with biomarkers of cardio-metabolic diseases among middle-aged and elderly Chinese.

**Methods:**

Survey of 1,458 men and 1,831 women aged 50 to 70 years, conducted in one urban and two rural areas of Beijing and Shanghai in 2005. SRH status was measured and categorized as good (very good and good) vs. not good (fair, poor and very poor). Determinants of SRH and associations with biomarkers of cardio-metabolic diseases were evaluated using logistic regression.

**Results:**

Thirty two percent of participants reported good SRH. Males and rural residents tended to report good SRH. After adjusting for potential confounders, residence, physical activity, employment status, sleep quality and presence of diabetes, cardiovascular disease, and depression were the main determinants of SRH. Those free from cardiovascular disease (OR 3.68; 95%CI 2.39; 5.66), rural residents (OR 1.89; 95% CI 1.47; 2.43), non-depressed participants (OR 2.50; 95% CI 1.67; 3.73) and those with good sleep quality (OR 2.95; 95% CI 2.22; 3.91) had almost twice or over the chance of reporting good SRH compared to their counterparts. There were significant associations -and trend- between SRH and levels of inflammatory markers, insulin levels and insulin resistance.

**Conclusion:**

Only one third of middle-aged and elderly Chinese assessed their health status as good or very good. Although further longitudinal studies are required to confirm our findings, interventions targeting social inequalities, lifestyle patterns might not only contribute to prevent chronic morbidity but as well to improve populations' perceived health.

## Background

Self-rated health (SRH) is a simple but comprehensive self-assessment of global (physical and mental) health status in which respondents are asked to rate their overall health, usually in a 5-point scale [1-3]. In previous studies, in a wide variety of populations, SRH has been demonstrated to be an accurate reflection of a person's health and a valid predictor of incident mortality and chronic morbidity (e.g. depression, diabetes) [3-10] that might be just as good as collecting extensive biological data [3,9]. Yu et al. [10] found that poor perceived health increased the relative risk of death by almost twice compared to excellent/good health among urban Shanghai residents. Leung et al. [8] studying elderly Chinese found a six time higher relative risk of death for those with poor levels of SRH (compared to good/excellent). Nevertheless, comprehensive assessments of the determinants of SRH among Chinese populations are scarce. Only a few studies [2,11-14] have evaluated the levels of SRH among Chinese but they only consider a single demographic nucleus, ignoring the potential effect of geographic location and residential status in a vast and populous country like China. Other factors such as sleep patterns and depression which might also significantly influence SRH have not been extensively investigated in the existing studies [2,11-14]. Furthermore just a few studies [15-17], none in mainland China, have evaluated whether SRH besides being a predictor of mortality and morbidity, could be associated with biomarkers of cardio-metabolic disorders among healthy populations and thus reflect early deviations from healthy trajectories.

We aimed to evaluate: 1) the distribution of SRH, among middle-aged and elderly populations in China; 2) the factors that determine SRH in this population; and 3) whether lower SRH is associated with worse levels of biomarkers of cardio-metabolic disease.

## Methods

### Study population

The current study is part of the "Nutrition and Health of Aging Population in China" study [18]. To provide a representative sample of elderly people in China, a cross-sectional survey of 3289 subjects (1,458 men and 1,831 women) aged 50 to 70 years from one urban and two rural areas of Beijing and Shanghai (cities of north and south of China) was conducted from March to June 2005. Streets or villages were randomly selected. Then participants were randomly selected from the eligible candidates listed in the residential registration record who were stable residents for 20 years in the area and were free from the following condition: 1) severe psychological disorders, physical disabilities, cancer, CVD, Alzheimer's disease or dementia, within 6 months; or 2) currently diagnosed with tuberculosis, AIDS and other communicable disease. The sampling was conducted with an emphasis on enlisting at least 40% of men, a similar number of rural/urban residents of Beijing and Shanghai and with representation from all levels of education and income. From a residency registration list in the selected streets or villages, 3533 potential participants were identified and interviewed in their households. The total response rate was 93.1% with no difference between the cities (93.5% in Beijing and 92.7% in Shanghai). A face to face interview with one respondent per household was completed by trained physicians or public health workers using a standardized questionnaire. 3379 persons agreed to participate in the study while 3289 eligible participants with complete information from questionnaire, physical examination and biomarkers were included in the final analysis. Self-rated health status and extensive socio-demographical, lifestyle and chronic disease information were collected from the participants. The study was approved by the Institutional Review Board of Institute for Nutrition Sciences, and informed consent was obtained from each participant. Further details about the questionnaire can be found elsewhere [19].

SRH was initially recorded in 5 levels (very good, good, fair, poor, and very poor), and then categorized into two categories good (very good and good) and poor (fair, poor and very poor) [2,13].

Socio-demographic variables included age (categorized as 50-59 and 60-70 yrs), gender, geographic region (Beijing/Shanghai), residential area (urban/rural), marital status (defined as married or without spouse (single, separated or widow)) and living status (defined as living alone versus with others). Socio-economic variables included educational attainment (categorized into low, moderate and high, based on the number of years of education as 0-6, 7-9, and ≥10 years respectively), employment status (grouped as currently employed, unemployed/on welfare system/retired) and total household income (categorized as low, moderate and high on the basis of <10,000, 10,000-29,999 and > = 30,000 RMB annually).

Lifestyle variables included social activity (defined as "yes"/"no" on the basis of frequently participating in at least one of social activities such as painting, playing chess, singing and dancing), smoking habit [defined as never, current (daily smoking, >6 months) and former (cessation of smoking >6 months)]; alcohol drinking ("yes "/"no"); physical activity level (classified as low, moderate, or high according to the guidelines for data processing and analysis of the International Physical Activity Questionnaire [5,20]); sleep quality during the last month (well, common or poor); and sleep quantity: average total hours of daily sleep during last month (categorized as under, normal and over (<7, 7 to 9, and >9 hours per day, respectively)).

Body mass index (BMI) levels were categorized as underweight (<18.5 kg/m2), normal weight (18.5 to <24.0 kg/m2), overweight (24.0 to <28.0 kg/m2) and obese (≥28.0 kg/m2)[21].

Medical insurance defined as "yes"/"no" on the basis of benefiting from one type of medical insurance such as social, commercial or governmental paid.

Three measurements of blood pressure were taken using an electronic blood pressure monitor and the mean of the last two measurements was used for analysis. Hypertension was defined if either the systolic blood pressure (SBP) is greater than 140 mmHg or diastolic blood pressure (DBP) is greater than 90 mmHg or taking anti-hypertensives. Presence of diabetes was defined as either measured fasting glucose greater than 7.0 mmol/L or being diagnosed with type-2 diabetes or taking anti-diabetic drugs or insulin. Presence of cardiovascular disease (CVD) was defined as the presence of one or more definite manifestations of coronary heart disease or stroke. Metabolic syndrome defined according to the updated National Cholesterol Education Program Adult Treatment Panel III (ATP III) criteria for Asian Americans[19,22].

To measure the presence of clinically relevant depressive symptoms, we used the Center for Epidemiologic Studies Depression Scale (CES-D) [23], which has been previously validated in Chinese populations [24,25]. Using a generally accepted cutoff point of 16 for the sum of scores, CES-D score of 0-15 was defined as no or minimal depression and ≥16 was defined as minor depression [23,26] to be consistent with the previous publications[18,27,28].

### Laboratory methods

In order to evaluate the association between SRH and cardio-metabolic disorders (e.g. cardiovascular disease, metabolic syndrome, diabetes mellitus) we measured a number of biomarkers in our population. Overnight fasting blood samples were collected in tubes containing liquid ethylenediaminetetraacetic acid (EDTA), centrifuged at 4°C, and stored at -80°C until analysis. Total cholesterol, high-density lipoprotein (HDL) cholesterol, low-density lipoprotein (LDL) cholesterol, triglycerides (TG), and glucose were measured enzymatically on an automatic analyzer (Hitachi 7080, Japan) with reagents purchased from Wako Pure Chemical Industries (Osaka, Japan). Plasma C-reactive protein (CRP) was measured by a particle-enhanced immunoturbidimetric assay (Ultrasensitive CRP kit, Orion Diagnostica, Espoo, Finland) using microparticles coated with anti-human CRP antibodies. Retinol-binding protein 4 (RBP4) was measured by a sandwich ELISA developed in-house, utilizing affinity-chromatography purified polyclonal and monoclonal antibodies generated against recombinant human RBP4, more details can be found elsewhere [29]. Interleuikin-6 (IL-6) was measured with high-sensitivity enzyme-linked immuno-sorbent assay (ELISA) (Quantikine HS IL-6 Immunoassay, R&D Systems, Inc., Minneapolis, Minnesota) with a low detect limit of 0.04 pg/ml.

Plasma adiponectin, resistin and active plasminogen activator inhibitor-1 (PAI-1) concentrations were measured by Luminex xMAPTM Technology (Linco Research Inc, St Charles, Mo) on a Bio-Rad Multiplex Suspension Array System (more details can be found elsewhere [18]. Fasting insulin was determined by radioimmunoassay (Linco Research, MO). Index of insulin resistance was calculated using updated homeostasis model assessment methods (HOMA2-IR, using the HOMA2 calculator, ) [30].

### Statistical analyses

First, we evaluated the distribution of SRH by gender, geographic region and area of residence. Then using the chi-square test, analysis of variance or Wilcoxon rank-sum test, we evaluated the associations between SRH and potential determinants, based on previous literature (Table 1). Factors that significantly modified SRH in the univariate analysis or that were consistently reported as significant determinants in previous publications were included in multivariate analysis (Table 2).

**Table 1:** Characteristics of the study participants by self-rated health status*

**Variable**	**Total** **(n = 3287)**	**Good** **(n = 1051)**	**Fair/Poor** **(n = 2236)**	***P *Value**
Age, mean (SD), y	58.6 (6.0)	58.00(5.99)	58.89(6.00)	< 0.0001
Age category, yrs				< 0.0001
50-59	1855(56.40)	651 (61.94)	1203 (53.80)	
60-70	1433 (43.60)	400 (38.06)	1033 (46.20)	
Female gender	1831 (55.70)	530 (50.43)	1301 (58.18)	< 0.0001
Beijing region	1640 (49.89)	489 (46.53)	1151 (51.48)	0.0081
Rural residence	1649 (50.17)	672 (63.94)	977 (43.69)	< 0.0001
Married currently	2880 (87.62)	941 (89.53)	1939 (86.72)	0.0222
Living status (alone, n(%))	186 (5.66)	50 (4.76)	136 (6.09)	0.1245
Educational level, years in school				< 0.0001
0-6	1360 (41.38)	509 (48.43)	851 (38.06)	
7-9	1172 (35.66)	354 (33.68)	818 (36.58)	
≥10	755 (22.97)	188 (17.89)	567 (25.36)	
Annual income, Yuan				0.0015
<10000	908 (29.04)	335 (33.27)	573 (27.03)	
10000-29999	1422 (45.47)	434 (43.10)	988 (46.60)	
≥30000	797 (25.49)	238 (23.63)	559 (26.37)	
Employment				< 0.0001
employed	784 (23.85)	336 (31.97)	448 (20.04)	
retired	1819 (55.34)	465 (44.24)	1354 (60.55)	
unemployed/on welfare	684 (20.81)	250 (23.79)	434 (19.41)	
Social activities (YES, n(%))	1670 (50.81)	541 (51.47)	1129 (50.49)	0.5991
Smoking				< 0.0001
current	918 (27.93)	362 (34.44)	556 (24.87)	
former	329 (10.01)	94 (8.94)	235 (10.51)	
never	2040 (62.06)	595 (56.61)	1445 (64.62)	
Current alcohol drinker	938 (28.54)	349 (33.21)	589 (26.34)	< 0.0001
Physical activity, level				< 0.0001
low	245 (7.45)	63 (5.99)	182 (8.14)	
moderate	1379 (41.95)	343 (32.64)	1036 (46.33)	
high	1663 (50.59)	645 (61.37)	1018 (45.53)	
Sleep quality				< 0.0001
well	1745 (53.20)	761 (72.55)	984 (44.11)	
common	1002 (30.55)	204 (19.45)	798 (35.77)	
poor	533 (16.25)	84 (8.01)	449 (20.13)	
Sleep quantity, total hours of sleep/day				< 0.0001
<7	809 (24.66)	201 (19.22)	608 (27.22)	
7-9	2211 (67.41)	769 (73.52)	1442 (64.55)	
>9	260 (7.93)	76 (7.27)	184 (8.24)	
Medical insurance (YES, n(%))	2297 (70.09)	693 (66.13)	1604 (71.96)	0.0007
Waist circumference(cm), mean (SD)	83.74 (10.55)	82.89 (10.06)	84.13 (10.75)	0.0017
Hip circumference(cm), mean (SD)	93.61 (6.85)	92.91 (6.48)	93.94 (7.00)	< 0.0001
BMI, mean (SD)	24.46(3.59)	24.20(3.39)	24.58 (3.67)	0.0053
BMI category				0.0061
under weight	111 (3.38)	37 (3.52)	74 (3.31)	
normal	1423 (43.29)	495 (47.10)	928 (41.50)	
over weight	1266 (38.52)	389 (37.01)	877 (39.22)	
obese	487 (14.82)	130 (12.37)	357 (15.97)	
Hypertension (YES, n(%))	1794 (54.58)	493 (46.91)	1301 (58.18)	< .0001
Diabetes (YES, n(%))	446 (13.95)	79 (7.68)	367 (16.92)	< 0.0001
CVD (YES, n(%))	333 (10.36)	27 (2.61)	306 (14.04)	< 0.0001
Metabolic syndrome (YES, n(%))	1401 (42.62)	352 (33.49)	1049 (46.91)	< 0.0001
Crude CES-D scores, mean (SD)	5.07(7.88)	2.89(5.36)	6.09(8.62)	< 0.0001
Depressive Symptoms (YES, n(%))	311 (9.46)	38 (3.62)	273 (12.21)	< 0.0001

Abbreviations: SD, standard deviation; CES-D, Center for Epidemiologic Studies Depression Scale; BMI, Body Mass Index; CVD, Cardio-Vascular Disease; CI, Confidence interval.

*Data are presented as frequency (percentage) unless otherwise indicated.

**There are two missing values for SRH and therefore the total number of 3287 subjects were included in the analysis**.

**Table 2:** Multivariate adjusted determinants of self-rated health status (good/poor) for the total population and stratified population by residential location or gender

	**Association with self rated health status (OR with 95% CI)**				
	
	**Total** **(n = 3289)**	**Rural** **(n = 1649)**	**Urban** **(n = 1640)**	**Female** **(n = 1831)**	**Male** **(n = 1458)**
Gender (female *vs*. male)	1.03 (0.79-1.33)	1.38 (0.96-2.00)	0.84 (0.57-1.23)		
Residential location (rural *vs. *urban)	1.89 (1.47-2.43)*			1.81 (1.25-2.63)*	2.00 (1.40-2.86)*
Geographic location (Beijing *vs. *Shanghai)	1.11 (0.92-1.33)	0.77 (0.59-1.02)	1.45 (1.10-1.91)*	0.92 (0.71-1.21)	1.22 (0.93-1.59)
Age group ‡ (group 1 *vs*. 2)	1.08 (0.89-1.32)	1.29 (0.99-1.68)	0.85 (0.62-1.18)	1.20 (0.92-1.57)	1.07 (0.78-1.48)
Marital status (spouse *vs. *no spouse)	1.11 (0.84-1.46)	1.20 (0.83-1.72)	0.91 (0.59-1.42)	1.30 (0.91-1.85)	0.84 (0.53-1.33)
Educational level, years in school †				*	
group 1 *vs. *3	1.21 (0.90-1.63)	0.76 (0.37-1.56)	1.17 (0.75-1.83)	1.87 (1.20-2.90)*	0.83 (0.53-1.28)
group 2 *vs. *3	1.03 (0.80-1.33)	0.73 (0.36-1.48)	1.14 (0.85-1.53)	1.56 (1.06-2.31)*	0.76 (0.53-1.10)
Annual income					
high *vs. *low	1.38 (1.05-1.82)*	1.40 (0.94-2.09)	1.23 (0.70-2.15)	1.32 (0.91-1.93)	1.39 (0.92-2.09)
medium *vs. *low	1.17 (0.94-1.46)	1.18 (0.92-1.52)	1.02 (0.60-1.76)	1.11 (0.82-1.50)	1.16 (0.84-1.60)
Employment	*				
employed *vs. *retired	1.36 (1.08-1.71)*	1.37 (1.01-1.85)*	1.37 (0.94-1.99)	1.36 (0.96-1.93)	1.35 (0.96-1.90)
unemployed *vs*. retired	1.26 (0.99-1.61)	1.29 (0.96-1.74)	0.86 (0.46-1.58)	1.32 (0.94-1.85)	1.13 (0.76-1.69)
Smoking				*	
current *vs. *never smoker	1.16 (0.89-1.51)	1.17 (0.81-1.68)	1.33 (0.90-1.96)	1.85 (1.11-3.10)*	1.00 (0.73-1.37)
former *vs. *never smoker	0.90 (0.63-1.27)	0.96 (0.60-1.54)	0.96 (0.55-1.66)	1.59 (0.69-3.66)	0.77 (0.52-1.15)
Alcohol drinking (yes *vs. *no)	1.26 (1.02-1.57)*	1.64 (1.21-2.23)*	1.03 (0.75-1.41)	1.10 (0.73-1.66)	1.35 (1.04-1.75)*
Physical activity	*		*	*	*
high *vs. *low	1.64 (1.16-2.33)*	1.56 (1.03-2.36)*	1.94 (0.99-3.79)	1.63 (1.02-2.59)*	1.76 (1.03-3.01)*
moderate *vs*. low	1.23 (0.86-1.78)	1.59 (1.01-2.51)*	1.14 (0.58-2.22)	1.26 (0.78-2.05)	1.29 (0.73-2.26)
Sleep quality	*	*	*	*	*
well *vs. *poor	2.95 (2.22-3.91)*	3.52 (2.41-5.16)*	2.26 (1.49-3.45)*	3.10 (2.17-4.44)*	2.77 (1.73-4.43)*
common *vs. *poor	1.11 (0.82-1.50)	1.27 (0.84-1.93)	0.90 (0.57-1.41)	1.12 (0.76-1.65)	1.08 (0.65-1.80)
Hypertension (no vs. yes)	1.39 (1.17-1.66)*	1.37 (1.08-1.73)*	1.39 (1.07-1.82)*	1.23 (0.96-1.56)	1.63 (1.27-2.09)*
Diabetes (no vs. yes)	2.03 (1.53-2.69)*	1.65 (1.12-2.42)*	2.87 (1.85-4.43)*	1.76 (1.17-2.65)*	2.36 (1.59-3.50)*
CVD (no *vs. *yes)	3.68 (2.39-5.66)*	5.87 (2.85-12.09)*	2.80 (1.63-4.81)*	2.92 (1.62-5.27)*	4.94 (2.57-9.48)*
Depressive Symptoms (no *vs. *yes)	2.50 (1.67-3.73)*	2.71 (1.64-4.49)*	1.93 (1.01-3.71)*	3.26 (1.90-5.59)*	1.70 (0.90-3.18)

Abbreviations: CES-D, Center for Epidemiologic Studies Depression Scale; CVD, Cardio-Vascular Disease; OR, Odds ratio; CI, Confidence interval.

*Statistically significant at a P-value level below 0.05.

† Group 1, 2 and 3 for educational level are 0-6, 7-9 and ≥10 years in school respectively.

‡ Group 1, 2 for age are 50-<60 and 60-70 years old respectively.

All analyses were repeated stratifying by gender, region and residence one at a time and by stratifying for two of these factors simultaneously.

The associations between SRH and biomarkers of cardio-metabolic disease were tested by parametric t-test (after logarithmic transformation of the data if deemed necessary to satisfy normality assumptions), or/and non-parametric Wilcoxon rank-sum test. Biomarkers included were: resistin, PAI-1, RBP4, adiponectin, IL-6, CRP, insulin, HOMA-IR, HOMA2-IR, SBP, DBP, HDL cholesterol, LDL cholesterol, total cholesterol and triglycerides. These analyses were performed on the total population and on a sub-sample of participants that were free from CVD, cancer and diabetes.

Statistical analyses were performed with the SAS statistical package version 9.1 (SAS Institute, Cary, NC). Statistical tests were two-sided and at the 5% level of significance.

## Results

### Characteristics of the participants

The mean age of the participants was 58.6. There were more women than men (55.7% vs. 44.3%) (Table 3). Participants were mostly married (88%), more than half of the participants were retired, 51% reported to be actively involved in social activities and 53% to have a good sleep quality. Although the majority of the participants were non-smokers (72.1% total, 95% women and 44% men), reported not to drink alcohol (71.4% total, 90% women and 48% men) and had moderate or high levels of physical activity (92.5% total, 92% women and 93% men), more than half of the population were overweight or obese (53.4% total, 56% women, 50% men). Nine percent of participants had depressive symptoms, 14% diabetes, 10% CVD, 55% hypertension and 43% had metabolic syndrome (Table 3).

**Table 3:** Characteristics of the study participants by residential location*

**Variable**	**Total** **(n = 3289)**	**Rural** **(n = 1649)**	**Urban** **(n = 1640)**	***P *Value**
Self-rated health status				< 0.0001
excellent and good	1051 (31.97)	672 (40.75)	379 (23.14)	
fair	1733 (52.72)	700 (42.45)	1033 (63.06)	
poor and very poor	503 (15.30)	277 (16.80)	226 (13.80)	
Age, mean (SD)	58.6 (6.0)	58.4 (5.8)	58.8 (6.2)	0.01
Age category, yrs				
50-59	1855 (56.40)	958 (58.10)	897 (54.70)	0.0492
60-70	1434 (43.60)	691 (41.90)	743 (45.30)	
Female gender	1831 (55.67)	906 (54.94)	925 (56.40)	0.3994
Beijing region	1641 (49.89)	812 (49.24)	829 (50.55)	0.4536
Married currently	2882 (87.63)	1436 (87.08)	1446 (88.17)	0.3436
Living status (alone, n (%))	186 (5.66)	104 (6.31)	82 (5.00)	0.1056
Educational level, years in school				< .0001
0-6	1360 (41.35)	1107 (67.13)	253 (15.43)	
7-9	1172 (35.63)	497 (30.14)	675 (41.16)	
≥10	757 (23.02)	45 (2.73)	712 (43.41)	
Annual income, Yuan				< .0001
<10000	909 (29.05)	790 (50.74)	119 (7.57)	
10000-29999	1422 (45.45)	613 (39.37)	809 (51.46)	
≥30000	798 (25.50)	154 (9.89)	644 (40.97)	
Employment				< 0.0001
employed	785 (23.87)	517 (31.35)	268 (16.34)	
retired	1819 (55.31)	556 (33.72)	1263 (77.01)	
unemployed/on welfare	685 (20.83)	576 (34.93)	109 (6.65)	
Social activities (Yes, n (%))	1671 (50.81)	626 (37.96)	1045 (63.72)	< 0.0001
Smoking				< .0001
current	919 (27.94)	513 (31.11)	406 (24.76)	
former	329 (10.00)	180 (10.92)	149 (9.09)	
never	2041 (62.06)	956 (57.97)	1085 (66.16)	
Current alcohol drinker	940 (28.58)	443 (26.86)	497 (30.30)	0.03
Physical activity, level				< .0001
low	245 (7.45)	161 (9.76)	84 (5.12)	
moderate	1381 (41.99)	400 (24.26)	981 (59.82)	
high	1663 (50.56)	1088 (65.98)	575 (35.06)	
Sleep quality				< 0.0001
well	1746 (53.20)	959 (58.26)	787 (48.11)	
common	1003 (30.56)	422 (25.64)	581 (35.51)	
poor	533 (16.24)	265 (16.10)	268 (16.38)	
Sleep quantity, total hours of sleep/day				< 0.0001
<7	810 (24.68)	390 (23.68)	420 (25.69)	
7-9	2212 (67.40)	1089 (66.12)	1123 (68.69)	
>9	260 (7.92)	168 (10.20)	92 (5.63)	
Waist circumference(cm), mean (SD)	83.74 (10.55)	82.61 (10.73)	84.87 (10.24)	< 0.0001
Hip circumference(cm), mean (SD)	93.61 (6.85)	91.91 (6.40)	95.32 (6.86)	< 0.0001
BMI, mean (SD)	24.46 (3.59)	24.05 (3.62)	24.88 (3.51)	< 0.0001
BMI category				< 0.0001
under weight	111 (3.37)	74 (4.49)	37 (2.26)	
normal	1423 (43.27)	788 (47.79)	635 (38.72)	
over weight	1268 (38.55)	570 (34.57)	698 (42.56)	
obese	487 (14.81)	217 (13.16)	270 (16.46)	
Medical insurance (Yes, n (%))	2299 (70.11)	768 (46.69)	1531 (93.70)	< .0001
Hypertension (Yes, n (%))	1796 (54.61)	930 (56.40)	866 (52.80)	0.0385
Diabetic (Yes, n (%))	447 (13.97)	184 (11.37)	263 (16.64)	< 0.0001
CVD (Yes, n (%))	333 (10.35)	125 (7.69)	208 (13.07)	< 0.0001
Metabolic syndrome (Yes, n (%))	1402 (42.63)	625 (37.90)	777 (47.38)	< 0.0001
Crude CES-D scores, mean (SD)	5.07(7.88)	5.27(8.41)	4.87(7.30)	0.40
Depressive Symptoms (Yes, n (%))	312 (9.49)	169 (10.25)	143 (8.72)	0.1346

Abbreviations: SD, standard deviation; CES-D, Center for Epidemiologic Studies Depression Scale; BMI, Body Mass Index; CVD, Cardio-Vascular Disease; CI, Confidence interval.

*Data are presented as frequency (percentage) unless otherwise indicated.

### Distribution of SRH

One third of the study population reported their health status as good or very good (32%), almost half of the population reported their health as fair (53%) and the rest as poor or very poor (15%) (Table 3).

Sixty-four percent of those that reported good SRH were rural residents. Those with poor SRH were more likely to be retired compared to those with good SRH (60.5% vs. 44.2%). Lower prevalence of high physical activity (45.5% vs. 61.4%) and good sleep quality (44.1% vs. 72.5%) and higher prevalence of CVD (14% vs. 2.6%), diabetes (16.9% vs. 7.7%) and depressive symptoms (12.2% vs. 3.6%) was observed among those that reported poor SRH compared to their counterparts (Table 1).

### Associated factors with Self-Rated Health

Based on univariate analysis (results not shown, only p-values presented in the Table 1), males, younger people (50-60 yrs), rural residents and those living in Shanghai were more likely to have a good SRH. Single (without spouse) individuals, retired and those with higher level of education or annual income are less likely to report a good SRH. Physically active people, smokers and drinkers had higher odds to report a good level of SRH. Also benefiting from a good sleep quality was significantly associated with a good level of SRH. Depressed, CVD and diabetic participants and those with hypertension and metabolic syndrome were more likely to have a poor SRH. Social activity levels and living status showed no association with SRH in the univariate analysis and were therefore not included in the multivariate analyses.

After adjusting for all selected factors in the multivariate analysis, living in rural area (Odds Ratio (OR) 1.89; 95% Confidence Interval (CI)1.47-2.43), being employed (OR 1.36; 95% CI 1.08-1.71), drinking alcohol (OR 1.26; 95% CI 1.02-1.57), having high levels of physical activity (OR 1.64; 95% CI 1.16-2.33) and benefiting from good sleep quality (OR 2.95; 95% CI 2.22-3.91) increased the chance of reporting a good SRH. Furthermore, being free of diabetes (OR 2.03; 95% CI 1.53-2.69), hypertension (OR1.39; 95% CI 1.17-1.66), CVD (OR 3.68; 95% CI 2.39-5.66) and depression (OR 2.50; 95% CI 1.67-3.73) independent of the other determinants significantly increased the chance of feeling healthier (Table 2).

Similar findings were obtained when the analyses were stratified by residence (urban vs. rural) or gender. Quality of sleep, diabetes and CVD were found significantly associated with SRH for all subgroups. We have also found significant association of depression (apart from male), hypertension (apart from female) and physical activity (apart from rural resident) in most of the subgroups. Among rural residents, drinking alcohol (OR 1.64; 95% CI 1.21-2.23) and among urban residents region of living (Beijing vs. Shanghai OR 1.45; CI 1.10-1.91) was significantly associated with SRH (Table 2). Educational levels and smoking (current vs. never OR 1.85; CI 1.11-3.10), would affect women's SRH while alcohol drinking (No vs. yes OR 0.74; CI 0.57-0.96) was associated with men's SRH (Table 2). Residence of living was found significant for both gender subgroups (female (rural vs. urban OR 1.81; CI 1.25-2.63), male (rural vs. urban OR 2.00; CI 1.40-2.86).

After stratifying the analyses by gender and residence of living simultaneously (Table 4), quality of sleep and CVD remained the most significant determinants of SRH for all subgroups. Hypertension affected males in both urban & rural areas but not females. Region of living and diabetes were significantly associated with SRH in rural women and urban men. Depressive symptoms were significantly associated with SRH for all subgroups except urban males. Educational level, employment status and level of physical activity were only associated with urban female's SRH. Age was significant between urban males and smoking between rural males.

**Table 4:** Multivariate adjusted determinants of self-rated health status (good/poor) stratified by residential location and gender (simultaneously)

	**Association with self rated health status (OR with 95% CI)**			
	
	**Rural-female** **(n = 925)**	**Rural-male** **(n = 715)**	**Urban-Female** **(n = 906)**	**Urban-Male** **(n = 743)**
Geographic location (Beijing *vs. *Shanghai)	0.59 (0.39-0.89)*	0.92 (0.62-1.37)	1.23 (0.83-1.81)	1.74 (1.16-2.63)*
Age group ‡ (group 1 *vs*. 2)	1.22 (0.85-1.75)	1.49 (0.98-2.28)	1.29 (0.83-2.02)	0.55 (0.32-0.95)*
Marital status (spouse *vs. *no spouse)	1.49 (0.92-2.42)	0.78 (0.43-1.42)	0.97 (0.55-1.70)	0.90 (0.42-1.94)
Educational level, years in school †			*	
group 1 *vs. *3	1.25 (0.31-5.08)	0.62 (0.25-1.56)	2.27 (1.24-4.17)*	0.53 (0.24-1.18)
group 2 *vs. *3	1.37 (0.34-5.63)	0.51 (0.21-1.26)	1.52 (0.99-2.35)	0.95 (0.61-1.47)
Annual income				
high *vs. *low	1.43 (0.82-2.49)	1.34 (0.74-2.42)	1.12 (0.53-2.37)	1.37 (0.57-3.27)
medium *vs. *low	1.23 (0.87-1.74)	1.11 (0.76-1.63)	0.93 (0.45-1.93)	1.11 (0.48-2.56)
Employment			*	
employed *vs. *retired	1.10 (0.71-1.70)	1.59 (0.99-2.55)	2.26 (1.25-4.08)*	1.36 (0.79-2.35)
unemployed *vs*. retired	1.21 (0.83-1.77)	1.36 (0.81-2.28)	0.55 (0.11-2.65)	1.08 (0.50-2.33)
Smoking	*			
current *vs. *never smoker	2.53 (1.27-5.04)*	0.80 (0.51-1.26)	1.45 (0.61-3.43)	1.37 (0.87-2.17)
former *vs. *never smoker	1.52 (0.50-4.66)	0.72 (0.41-1.25)	2.17 (0.58-8.12)	0.87 (0.47-1.62)
Alcohol drinking (yes *vs. *no)	1.15 (0.59-2.27)	1.76 (1.23-2.52)*	1.08 (0.63-1.85)	1.06 (0.70-1.58)
Physical activity			*	
high *vs. *low	1.39 (0.81-2.39)	2.01 (1.03-3.93)*	2.42 (0.90-6.52)	1.76 (0.67-4.59)
moderate *vs*. low	1.75 (0.97-3.17)	1.62 (0.77-3.39)	1.22 (0.46-3.25)	1.14 (0.44-2.99)
Sleep quality	*	*	*	*
well *vs. *poor	4.09 (2.54-6.58)*	2.53 (1.30-4.91)*	1.95 (1.14-3.34)*	3.04 (1.50-6.13)*
common *vs. *poor	1.49 (0.89-2.49)	0.95 (0.45-1.98)	0.73 (0.40-1.33)	1.16 (0.55-2.42)
Diabetic (no vs. yes)	1.83 (1.04-3.21)*	1.54 (0.88-2.67)	1.77 (0.95-3.29)	4.35 (2.30-8.22)*
Hypertension (no vs. yes)	1.14 (0.82-1.58)	1.74 (1.23-2.45)*	1.27 (0.87-1.86)	1.48 (1.00-2.18)*
CVD (no *vs. *yes)	3.55 (1.44-8.76)*	13.34 (3.11-57.28)*	2.51 (1.13-5.56)*	3.32 (1.56-7.05)*
Depressive Symptoms (no *vs. *yes)	2.69 (1.42-5.09)*	2.82 (1.17-6.83)*	4.14 (1.41-12.10)*	0.87 (0.35-2.16)

Abbreviations: CES-D, Center for Epidemiologic Studies Depression Scale; CVD, Cardio-Vascular Disease; OR, Odds ratio; CI, Confidence interval. * Statistically significant at a P-value level below 0.05.

† Group 1, 2 and 3 for educational level are 0-6, 7-9 and ≥10 years in school respectively.

‡ Group 1, 2 for age are 50-<60 and 60-70 years old respectively.

### SRH and cardio-metabolic biomarkers

Significant differences in level of biomarkers (except for adiponectin) were observed between the participants with different level of SRH. The level of SRH inversely related to the levels of resistin, CRP, insulin, HOMA-IR, HOMA2-IR, systolic and diastolic blood pressure (Table 5). Trends and significant differences remained for resistin, CRP, insulin and HOMA2-IR among a healthier subgroup of participants that was free from cancer, CVD and diabetes (data not shown).

**Table 5:** Cardio-metabolic risk factors and biomarkers of the study participants by self rated health status

**Variable, mean (SD)**	**Total** **(n = 3289)**	**Good** **(n = 1051)**	**Fair** **(n = 1733)**	**Poor** **(n = 503)**	***P *Value#**
Resistin	11.45(9.36)	11.03(9.44)	11.42(8.62)	12.49(11.37)	0.01208*
PAI-1	14.72(18.13)	13.31(17.45)	15.80(18.67)	13.83(17.21)	< 0.0001*
RBP4	40.1(11.75)	39.05(11.39)	40.74(11.84)	40.08(12.05)	0.0014*
Adipo	16.50(11.65)	16.06(11.49)	16.50(11.51)	17.41(12.38)	0.252
IL-6	1.55(2.77)	1.49(2.52)	1.45(2.29)	2.02(4.30)	0.0003*
CRP	1.57(4.29)	1.23(2.12)	1.66(5.22)	1.97(4.06)	< 0.0001*
Insulin	15.44(9.48)	14.27(9.13)	15.67(8.40)	17.12(12.83)	< 0.0001*
Homa-ir	4.09(3.35)	3.61(2.66)	4.18(3.08)	4.78(5.03)	< 0.0001*
Homa2-ir	0.30(0.22)	0.28(0.26)	0.31(0.17)	0.34(0.27)	< 0.0001*
SBP	140.12(22.47)	138.62(22.13)	139.72(22.36)	144.66(23.04)	< 0.0001*
DBP	80.17(10.80)	79.69(10.38)	80.05(11.02)	81.56(10.83)	< 0.005*
HDL	1.28(0.33)	1.31(0.34)	1.26(0.33)	1.27(0.34)	< 0.0018*
LDL	3.26(0.97)	3.14(0.94)	3.33(0.97)	3.28(1.00)	< 0.0001*
TCH	4.70(0.98)	4.58(0.94)	4.76(0.98)	4.71(1.03)	< 0.0001*
TG	1.39(1.07)	1.24(0.92)	1.48(1.17)	1.39(0.96)	< 0.0001*

Abbreviations: PAI_1, plasminogen activator inhibitor-1; RBP4, retinol binding protein 4; ADIPO, adiponectin; IL6, interleukin-6; IL6, interleukin-6; CRP, c-reactive protein; Homa-ir, homeostasis model assessment of insulin resistance (fasting glucose (mmol/L) × fasting insulin (μU/ml)/22.5); SBP, systolic blood pressure; DBP, diastolic blood pressure; HDL, high-density lipoprotein; LDL, low-density lipoprotein; TCH, total cholesterol; TG, triglycerides; SD, standard deviation;

# Wilcoxon/t-test p-value that indicates the difference of the levels of biomarkers within the levels of SRH; *P < 0.05

## Discussion

We found a relatively low level of good SRH among middle-aged and elderly Chinese (32%), which varied substantially by area of residence, lifestyle and socio-economic factors and presence of chronic diseases. Lower levels of SRH were associated with worsened levels of inflammatory markers, blood lipids, insulin and insulin resistance in both the total population and a sub sample free from CVD, diabetes and cancer.

Only one third of the elderly Chinese assessed their health as good or very good. Despite lower socio-economic status (lower average income, fewer years in full-time education and less medical insurance coverage), rural residents were almost twice as likely to feel healthier compared to urban residents. This could be the consequence of different lifestyle followed by those living in rural areas, characterized by higher levels of physical activity, lower BMI and a higher age of retirement. Furthermore, the prevalence of both CVD (7.7% rural vs. 13% urban) and diabetes (11.4% rural vs. 17% urban) was lower among rural residents (Table 3).

Compared to the previous studies, we found relatively lower level of good/excellent SRH. This could be due to differences in the selection criteria (age range, disease status, functional status, cognitive status, exclusion criteria, etc.) or the coverage of our sample.

In previous studies, Yu et al. [10] by studying 3094 elderly (over 65 yrs) Chinese living in urban Shanghai found a level of good/excellent SRH of approximately 49% (15% higher than our finding in the urban-shanghai). This difference could be due to the selection criteria used in the study. Moreover, Lim et al. [2] studied 6236 individuals aged 18 and above (76% under 50 yrs) in Singapore (urban area) and found a level of good/excellent SRH of 77% (54% higher than our findings for urban residents) and Pei et al. [13] by studying 9594 aged 18 and over Chinese, found a level of good SRH of 72%. The difference between our study and these two studies could be due to a high proportion of study participants below age 50 and therefore a lower prevalence of comorbidities and other factors that increase with age and significantly modify SRH. Consistent with our findings, Pei et al. [13] found a significant association between residential affiliation and SRH, suggesting that living in rural area decreases the chance of reporting poor SRH.

Similar to our findings, Leung et al. [8] by studying 411 elderly (65 and over) Chinese living in two long-term care institutions found a relatively lower level of good/excellent SRH of approximately 33%. This could be due to their selection criteria (retired government employees, single or widows or poor or homeless participants who needed care (55% suffered from 4 or more chronic illnesses)) and older age (65% over 75 years). Goldman et al. [15] also showed the level of good SRH of approximately 25% in elderly Taiwan residents which could be due to the older sample population. Different levels of good SRH have also been reported for other countries such as USA [16] (66%), and Norway [17] (65%).

We found a significant association between different lifestyle characteristics and SRH in the current analyses. High levels of physical activity were associated with better SRH. Sleep quality was one of the factors which had the highest level of association with SRH in the study population. Those with good sleep quality were almost twice likely to report good levels of SRH. This result was consistent for all subgroups (regional, residential and gender) of the study population. The effect of smoking and alcohol drinking on SRH could be hampered by the effect of gender as the majority of Chinese smokers and drinkers were men. Nevertheless, we found a significant association of alcohol intake and SRH on the total population and among rural residents. The association however indicated the higher chance of reporting good SRH for those drinking alcohol. This could be due to the lack of information about drinking habits (number of units or frequency of alcohol consumption) in the models.

Furthermore, socio-economic factors, such as education and employment status, affected mainly urban women. Most of the previous studies [12,13,31] indicated that lower levels of income or education were significantly associated with reporting poor SRH. Consistent with previous publications [12,31], we found a significant difference in the level of SRH of those reported high and low level of income in the total population but no association of income in any other subgroups of the study population. A significant association of education has only been found in females and urban females subgroups. This could be due to the fact that the area of residence might be a more comprehensive factor in our study population and might partially cover the effect of socio-economic factors (e.g. the Chinese population in our sample is skewed towards lower income and education and late retirement in rural areas).

Absence of CVD, hypertension, diabetes or depression increased the chance of reporting a good level of SRH. Presence of depressive symptoms had a bigger impact on women and urban residents. Suffering from CVD was found consistently associated with SRH levels for all subgroups of gender and area of residence with the strongest association among rural-men as more likely to report poor SRH. The deleterious association of CVD presence on SRH was higher among rural residents and men compared to urban residents and women. Consistent with our findings, by studying 1589 elderly Chinese in Hong Kong, Cheng et al. [11] found a strong association between SRH and chronic illnesses and sleep quality. Also Li et al. [1] completed a study on 56162 Chinese elderly in Hong Kong and found a significant association between time comparative SRH and active chronic disease and depression. Lim et al. [2] by studying 6236 individuals aged 18 and above in Singapore found a significant association between SRH and reported illnesses (cancer and CVD) and income.

Lower concentrations of inflammatory biomarkers, plasma insulin and index of insulin resistance were associated with good levels of SRH for the total population and among a subgroup of participants that were free from cancer, CVD and diabetes. Levels of SRH might effectively reveal early deviations from healthy trajectories, making SRH an effective assessment tool to evaluate healthy aging targeting the early prevention of the appearance of chronic disease. To our knowledge, no study has been conducted on Chinese in mainland China to examine the association between cardio-metabolic biomarkers and SRH. Only few studies investigated this aspect. For instance, consistent with our findings, Tomten et al. [17] studied 18770 Norwegians and found increasing HDL raised the odds of reporting good SRH. Jylha et al. [16] studied 4065 elderly people aged 71 and over in the USA and found a significant association between biomarkers (blood levels of albumin, hemoglobin, HDL cholesterol and white cell count) and SRH after adjusting for age and sex. Moreover, Goodman et al. [15] studied 928 respondents aged 54 and over in Taiwan and found a significant association between SRH and clinical variables. The strongest association was found for HDL cholesterol among men even in the presence of other control variables.

This is as well the first study to ever evaluate the associations between SRH and inflammatory markers and measures of insulin resistance in any population and the first study to evaluate the levels and distribution of SRH, its determinants and its associations with biomarkers of cardio-metabolic disease among Chinese populations from different geographical areas and residence.

We considered the effect of comprehensive measurements of lifestyle, socio-demographic factors and presence of disease in the analysis. The extrapolation of our findings is somewhat limited due to the cross-sectional nature of our data and further longitudinal studies are required to confirm our findings. Additionally no clinical diagnostic was carried out to validate the result and the severity of the diseases. Also the functional and/or cognitive level of participants, which has been previously found to be associated with SRH [5], was not included in the analysis. Finally, although the sampling was conducted to represent the whole population of middle-aged/elderly Chinese, due to the limited number of selected sites (3 in each city) from only two major cities (Beijing and Shanghai) of China, the total population of China was not represented completely, hence limiting the potential extrapolation of our findings to alternative populations.

## Conclusion

In general, we have found relatively low levels of good SRH in our population alongside with substantial impacts of area of residence, sleep quality and chronic diseases on SRH. Good SRH was associated with lower levels of inflammatory markers, insulin levels and insulin resistance, which might present SRH as a potential assessment tool to evaluate early deviations from health. Although further longitudinal studies are required to confirm our findings, interventions targeting social inequalities, sleep and physical activity patterns might not only contribute to prevent chronic morbidity but also to improve populations' perceived health.

## Abbreviations

BMI: body mass index; CI: confidence interval; CVD: cardiovascular disease; DBP: diastolic blood pressure; HDL: high-density lipoprotein; LDL: low-density lipoprotein; MetS: metabolic syndrome; OR: odds ratio; SBP: systolic blood pressure; SD: standard deviation; SRH: self-rated health; TG: triglycerides; TCH: total cholesterol; RBP4: retinol-binding protein 4; ADIPO: adiponectin; IL-6: interleukin-6; PAI-1: plasminogen activator inhibitor-1; CRP: C-reactive protein; ANOVA: analysis of variance

## Competing interests

The authors declare that they have no competing interests.

## Authors' contributions

NHM performed the statistical analysis, interpretation of data and sequence alignment and drafted the manuscript. AP participated in design of the study, data collection and manuscript revision. XY, JW, QQ, YL, HL, ZY participated in the design of the study and data collection. XL carried out in design of the study and supervised the study progress in china. OHF conceived the study and participated in the analysis, drafting and revising the manuscript. All authors read and approved the final manuscript.

## Pre-publication history

The pre-publication history for this paper can be accessed here:


